# Army Dental Practice vs. Civilian Dental Practice

**Published:** 1919

**Authors:** 


***Army Dental Practice vs. Civilian Dental Practice.*** By Lieut.-Col. John O. Lessig. *Journal Association Military Dental Surgeons of U. S.*, October 1918.

The author states that inspection of soldiers' oral cavities show " about ten per cent. in Class A, or good condition, seventy per cent. in Class B, or mouths with calculus surrounding teeth and several carious teeth, and twenty per cent. in Class C, very bad condition ".

The most important work of the army dentist is preventive. While there is a good deal of repair work to be done, it is most important to instruct the men in prophylaxis which will save many teeth. The army work is important because many men are given proper dental care and instruction in the care of the teeth for the first time.

